# The Transcription Factor Stp2 Is Important for *Candida albicans* Biofilm Establishment and Sustainability

**DOI:** 10.3389/fmicb.2020.00794

**Published:** 2020-04-30

**Authors:** Bettina Böttcher, Bianca Hoffmann, Enrico Garbe, Tobias Weise, Zoltán Cseresnyés, Philipp Brandt, Stefanie Dietrich, Dominik Driesch, Marc Thilo Figge, Slavena Vylkova

**Affiliations:** ^1^Septomics Research Center, Friedrich Schiller University and Leibniz Institute for Natural Product Research and Infection Biology – Hans Knöll Institute, Jena, Germany; ^2^Applied Systems Biology, Leibniz Institute for Natural Product Research and Infection Biology – Hans Knöll Institute, Jena, Germany; ^3^BioControl Jena, Jena, Germany; ^4^Institute of Microbiology, Faculty of Biological Sciences, Friedrich Schiller University Jena, Germany

**Keywords:** Biofilms, filamentation, *Candida albicans*, amino acids, metabolism, Stp2, automated image analysis

## Abstract

The fungal pathogen *Candida albicans* forms polymorphic biofilms where hyphal morphogenesis and metabolic adaptation are tightly coordinated by a complex intertwined network of transcription factors. The sensing and metabolism of amino acids play important roles during various phases of biofilm development – from adhesion to maturation. Stp2 is a transcription factor that activates the expression of amino acid permease genes and is required for environmental alkalinization and hyphal growth *in vitro* and during macrophage phagocytosis. While it is well established that Stp2 is activated in response to external amino acids, its role in biofilm formation remains unknown. In addition to widely used techniques, we applied newly developed approaches for automated image analysis to quantify Stp2-regulated filamentation and biofilm growth. Our results show that in the *stp2*Δ deletion mutant adherence to abiotic surfaces and initial germ tube formation were strongly impaired, but formed mature biofilms with cell density and morphological structures comparable to the control strains. Stp2-dependent nutrient adaptation appeared to play an important role in biofilm development: *stp2*Δ biofilms formed under continuous nutrient flow displayed an overall reduction in biofilm formation, whereas under steady conditions the mutant strain formed biofilms with lower metabolic activity, resulting in increased cell survival and biofilm longevity. A deletion of *STP2* led to increased rapamycin susceptibility and transcriptional activation of *GCN4*, the transcriptional regulator of the general amino acid control pathway, demonstrating a connection of Stp2 to other nutrient-responsive pathways. In summary, the transcription factor Stp2 is important for *C. albicans* biofilm formation, where it contributes to adherence and induction of morphogenesis, and mediates nutrient adaption and cell longevity in mature biofilms.

## Introduction

*Candida albicans* is the fungal species most frequently associated with the healthy human gastrointestinal, vaginal and skin microbiome. Under certain circumstances, such as immune suppression or disruptions of the associated microbiota, it can become pathogenic and infect virtually any part of the human body. Particularly problematic is its ability to adhere to catheters and indwelling medical devices, such as artificial heart valves and joint replacements, and proliferate to form biofilms (Nobile and Johnson, 2015). These highly antibiotic-resistant, complex cell communities can serve as a reservoir of infection, since detached biofilm cells can disseminate to multiple body sites (Uppuluri et al., 2018), resulting in life-threatening diseases like sepsis. In most cases, the removal of infected biomedical devices with auxiliary antibiotic administration remains the only effective treatment (Cornely et al., 2012). Even so, *Candida* spp. are consistently the third leading cause of device-associated bloodstream infections with mortality rate of up to 50% (Kojic and Darouiche, 2004).

The ability of *C. albicans* to undergo a morphogenetic transition from yeast to hyphae is critical for proper biofilm formation (Richard et al., 2005). Hyphae-associated factors, like the Als family of adhesins promote anchoring to a substratum, cell-cell adhesion, and are important for biofilm establishment particularly under fluid flow and mechanical shear force conditions (Nobile et al., 2008; Grubb et al., 2009; Finkel et al., 2012). Further, the elongated filaments serve as a scaffold in the mature biofilm, which is composed of a dense network of yeasts, hyphae, and pseudohyphae embedded into extracellular matrix. Biofilm-associated hyphal growth is regulated by seven key transcription factors, which, together with two regulators of glycolysis and carbon metabolism, comprise a tightly controlled intertwined network (Bonhomme et al., 2011; Cleary et al., 2012; Fox et al., 2015). Thus, both filamentation and fungal metabolism play a critical role in *C. albicans* biofilm formation. Indeed, increased biomass limits the diffusion of nutrients, oxygen and water, leading to constant metabolic adaptations. Previous reports describe dynamic transcriptional and proteomic rearrangements in glucose and amino acid metabolism, the tricarboxylic acid (TCA) cycle, and the respiratory chain (García-Sánchez et al., 2004; Lattif et al., 2008; Fox et al., 2015). High glucose levels support biofilm development by activating the yeast-to-hypha transition via the Ras/cAMP/PKA pathway (Sabina and Brown, 2009; Santana et al., 2013). *C. albicans* strains capable of producing robust biofilms are signified by specific upregulation of genes in different amino acid metabolic pathways, a process coordinated by the aspartate aminotransferase Aat1 (Rajendran et al., 2016).

In *C. albicans* two distinct, but partially interconnected regulatory systems control the sensing and uptake of amino acids. Environmental oligopeptides and amino acids are sensed via the SPS (Ssy1−Ptr3−Ssy5) signaling pathway, where the Ssy5 endoproteinase cleaves the N-terminal cytoplasm-retaining domain of the transcription factors Stp1 and Stp2, inducing the expression of oligopeptide transporter and amino acid permease-encoding genes, respectively (Martínez and Ljungdahl, 2005; Miramón and Lorenz, 2016). Sensing of intracellular amino acids is coordinated by the TOR-pathway, and nutrient starvation or a direct inhibition of the TOR complex by rapamycin blocks the signaling cascade and leads to de-repression of nitrogen catabolism (Lee et al., 2018).

The TOR pathway has broad functions in *C. albicans* where it influences virulence features including flocculation, filamentation, chlamydosporulation, and biofilm formation (Bastidas et al., 2009; Böttcher et al., 2016; Flanagan et al., 2017). Additional regulators also control both nitrogen metabolism and biofilm formation. Arg81, a transcription factor required for utilization of ornithine as a nitrogen source, is important for adherence under flow conditions (Finkel et al., 2012), whereas Gcn4, the key regulator of the general amino acid control (GAAC) pathway, is required for normal biofilm growth (García-Sánchez et al., 2004). While these reports demonstrate the extensive role for factors responding to intracellular amino acid levels in biofilm development, the importance of the SPS pathway in biofilm formation remain poorly characterized.

Here, we investigated the role of Stp2, the key transcriptional regulator of extracellular amino acid signaling and metabolism, in biofilm development. The results indicate that Stp2 regulates adherence and germ tube formation, which are essential for biofilm initiation. At later time points, *stp2*Δ strains formed robust biofilms with morphology and density comparable to the control strains. However, Stp2-defective mature biofilms displayed significantly reduced metabolic activity and prolonged survival. Therefore, Stp2-mediated metabolic adaptation supports the fundamental steps of *C. albicans* biofilm development and coordinates biofilm lifespan.

## Results

### Stp2 Is Required for Adherence to Abiotic Surfaces

**FIGURE 1:**
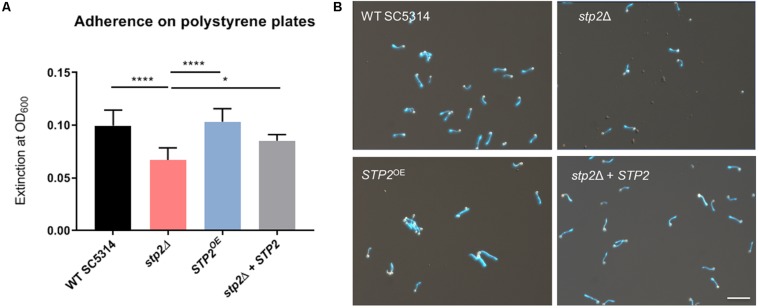
*C. albicans* Stp2 mediates adherence to polystyrene surfaces. *C. albicans* strains were incubated on 24 well polystyrene plates for 90 min in RPMI and non-adherent cells were washed off. **(A)** The density of the remaining cells was measured optically by OD_600 nm_. Statistical comparison revealed a significantly lower cell density for the *STP2* deletion strain to all the other strains (*n* = 3, One-way ANOVA with Tukey’s multiple comparisons test, *p* < 0.0001), indicating the importance of Stp2 in the process of adherence to abiotic surfaces. **(B)** Imaging of adherent cells after dual staining with rhodamine and calcofluor white. Microscopic analyses of *stp2*Δ showed a reduced cell number and shorten hyphae length. Scale bar: 50 μm.

Nutrient sensing and utilization is important for *C. albicans* filamentation and biofilm formation, and mature biofilms are linked to activation of the TCA cycle and amino acid metabolism. Since the transcription factor Stp2 controls utilization of extracellular amino acids, we investigated its role for biofilm establishment and maturation. Biofilms are initiated by adherence of individual cells to solid biotic or abiotic surfaces, so we tested the role of Stp2 in attachment to polystyrene culture plates. Cells lacking *STP2* had a significantly decreased ability to adhere to surfaces as compared to the wild type SC5314, *stp2Δ* + *STP2* and the *STP2*^OE^ strains (Figures 1A,B). *C. albicans* adherence is closely coupled to germ tube formation, and we noted that in addition to the impaired adherence *stp2*Δ cells had defect in filamentation (Figure 1B), which further contributed to the reduction in optical density. The overexpression of *STP2* did not promote cell adherence to the abiotic surface beyond the wild-type level. No growth defects were observed for the *stp2*Δ and *STP2*^OE^ strains compared to wild type and complemented strains in liquid RPMI medium (Supplementary Figure S1).

### Reduced Expression of Early Hyphae Aassociated Genes Correlates With Stp2 Processing

To gain further insight into the effect of Stp2 on hyphal morphogenesis, we established an automated method to quantify yeast-to-hyphae transition in shaking culture. For this purpose, the surface of mother yeast cells was stained with rhodamine, followed by incubation in RPMI medium at 37°C under shaking conditions to induce hyphal growth. After 90 min all cells, including budding yeasts and hyphal elements, were counterstained with calcofluor white and visualized by fluorescence microscopy. By automated image analysis, the hyphal filaments were segmented and separated from their mother yeast cells. Sample sizes of 83 to 100 elements per strain were examined for the number of germ tubes per yeast cell and the hyphal length.

**FIGURE 2:**
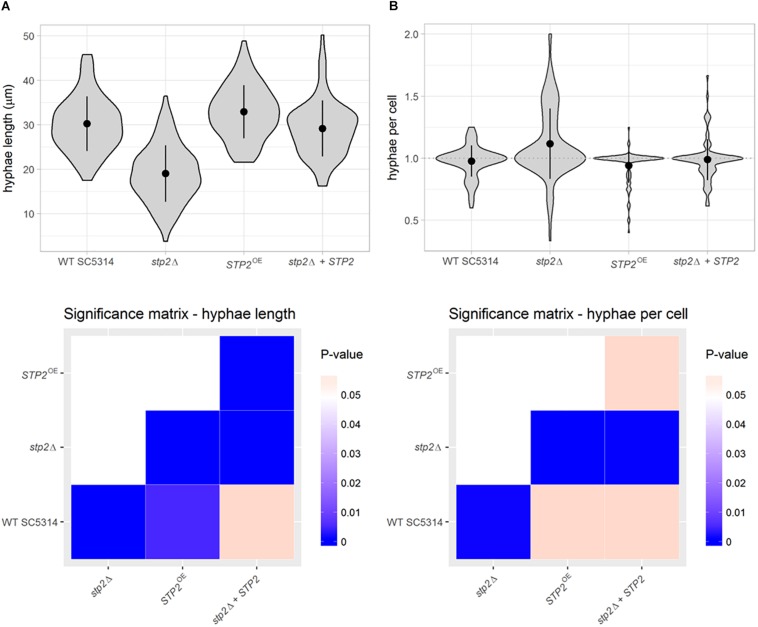
Stp2 contributes to filamentation in liquid RPMI medium. Filamentation of *C. albicans* strains was initiated in liquid medium for 90 min. Dual staining facilitated the differentiation of yeast and hyphae parts of the germinating cells. **(A)** Hyphal length in RPMI was determined using automated object identification and segmentation. The *stp2*Δ mutant and the *STP2* overexpression strain showed significantly shorter or longer hyphae, respectively, compared to the SC5314 and the stp2Δ + *STP2* strains. **(B)** The number of hyphae per mother cell was significantly higher for the *stp2*Δ mutant compared to the wild type, complemented and overexpression strains. P-values were obtained by pairwise Wilcoxon rank sum tests and corrected for multiple testing by the Benjamini-Hochberg procedure.

This novel approach revealed that the *stp2*Δ mutant formed significantly shorter filaments than the strains with a functional Stp2, with an average hyphae length of 19.05 μm, compared to 30.27 μm in wild type cells and 29.2 μm in the heterozygous *STP2* revertant (Figure 2A). Notably, *STP2* overexpression led to a significant increase in hyphae length compared to the wild type (32.96 μm; 8% longer). In addition, the number of hyphal filaments per mother cell was calculated and an average *stp2*Δ cell formed 1.12 germ tubes, resulting in a higher tendency to form multiple filaments per yeast cell, whereas the mean number of germ tubes was close to one for all other strains (Figure 2B).

**FIGURE 3:**
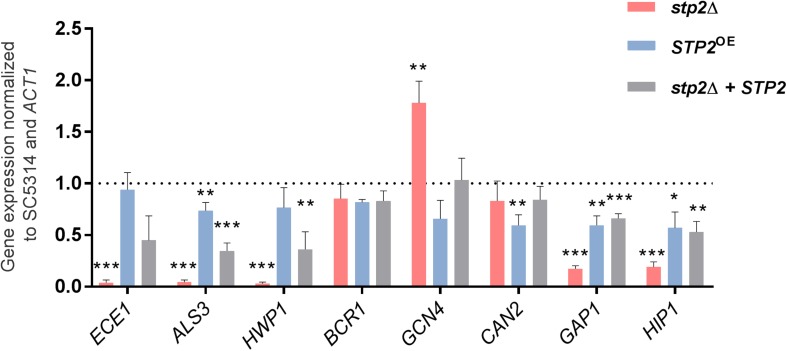
STP2-defective *C. albicans* strain has lower relative gene expression of hyphae-associated and amino acid permeases genes in RPMI medium. Early transcriptional response of cells grown in shaking, planktonic RPMI cultures after 60 min was analyzed using quantitative real time PCR. Two groups of genes were analyzed: the key morphology-associated genes *ECE1*, *ALS3, HWP1*, and *BCR1*; and amino acid and nitrogen metabolism genes *GCN4*, *CAN2*, *GAP1*, and *HIP1*. Depicted bars represent the relative fold expression change normalized to the housekeeping gene *ACT1* and the wild type SC5314 gene expression using the 2^– ΔΔ*CT*^ method (Pfaffl, 2001). Shown are means and standard deviations from three biological and three technical replicates and the dashed line represents the wild-type expression level. Statistical analyses compared each sample to the SC5314 wild type level (Multiple unpaired *t*-test, ***p < 0.001; **p < 0.005, and *p < 0.01; for samples that passed the False Discovery Rate approach by the Benjamini–Hochberg procedure with 1% threshold, *n* = 3). The *stp2*Δ mutant strain showed significantly reduced expression levels compared to the wild type SC5314 for all genes tested, but *CAN2*.

To test if the filamentation delay can be detected on transcriptional level, we analyzed the expression of early hyphae associated-genes *ECE1*, *ALS3* and *HWP1* by qRT-PCR. Their expression was significantly downregulated in the *stp2*Δ mutant compared to the SC5314 strain (Figure 3), showing that the altered filamentation is also reflected on gene expression level. Importantly, similar effects were also observed when cells were grown under strong Stp2-stimulating conditions, since *stp2*Δ cells had reduced expression of hyphae-associated genes (*ECE1*, *ALS3*, and *HWP1*) with a significant effect for *HWP1* (Supplementary Figure S2). *C. albicans stp2*Δ + *STP2* cells grown in RPMI medium restored the transcript level of early hyphal genes to an intermediate degree, and the *STP2* overexpression brought gene expression to the wild type level, except for expression of *ALS3*, which was significantly lower.

Genes for amino acid permeases (AAPs) are known targets of Stp2 and their enhanced transcription suggests active Stp2 processing. As expected, the impact of this transcription factor on AAP gene expression was apparent in casamino acid-rich (CAA) conditions, since we noted that the *stp2*Δ cells had significantly reduced expression of *CAN2*, *HIP1*, and most of the *GAP* genes (Supplementary Figure S2). In contrast, *GAP4* was upregulated suggesting that either additional transcriptional activator regulates the expression of this gene or that Stp2 acts as a repressor. We also tested AAPs gene expression in RPMI medium, condition that resulted in downregulation of *GAP1* and *HIP1* in cells lacking *STP2* compared to the wild type (Figure 3). However, the expression of these genes was still higher than for the hyphae-associated genes, further suggesting regulation by alternative or redundant transcription factors. Indeed, the expression of *GCN4*, a key regulator of nitrogen starvation responses, was enhanced in the *stp2*Δ mutant compared to the wild type. Counter-intuitively, the basic amino acid permease gene *CAN2* was not differentially regulated, and thus its gene expression was independent of Stp2 in the tested condition.

**FIGURE 4:**
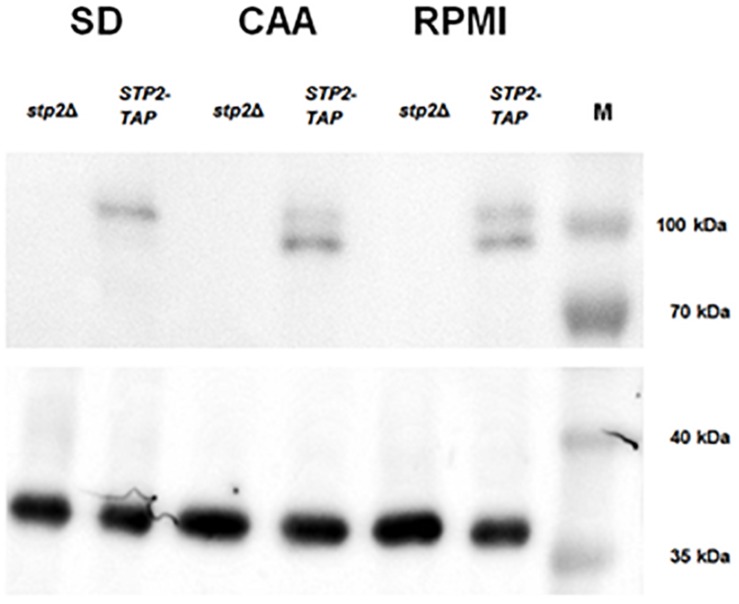
Growth in RPMI induces partial Stp2 processing. *STP2-TAP* and the parental *stp2*Δ strain were cultivated in liquid SD (4 h), CAA (4 h) or RPMI (90 min) media and protein processing was analyzed by Western blot. As expected, growth in the amino acid-free SD medium did not activate Stp2 processing, since only the full-length protein was detected. The amino acid-containing media CAA and RPMI induced N-terminal cleavage, as both the shorter and full-length Stp2 protein forms were detected. Tdh3 was used as a loading control and the PageRuler^TM^ protein ladder (10–180 kDa).

The activation of Stp2 is initiated by post-translational N-terminal cleavage, and removal of the cytoplasmic retention signal allows migration to the nucleus (Martínez and Ljungdahl, 2005). Since we noted Stp2-dependent expression of hyphal and AAPs genes in RPMI medium, we questioned whether these conditions stimulate Stp2 activation. Thus, processing of the Stp2 protein was analyzed after incubation in RPMI medium and compared to the conditions that stimulate Stp2 processing (CAA medium) or not (SD medium). As expected, Western Blot analyses of Stp2 processing revealed no cleavage of Stp2 in SD medium and nearly full processing in CAA. Partial processing of Stp2 was observed upon growth in RPMI medium (Figure 4). Thus, the amino acids available in this medium are sufficient to activate Stp2, leading to reduced expression of early hyphae-associated and AAP genes, and contributing to the notable filamentation defect in the *stp2*Δ mutant.

### Deletion of *STP2* Enhances the Starvation-Induced Hyphal Morphogenesis

**FIGURE 5:**
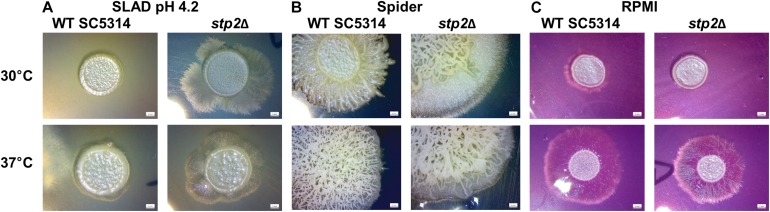
Stp2 controls filamentation on solid media depending on the available nutrient sources. 5 μl of *C. albicans* SC3514 or *stp2*Δ suspensions were dropped onto different nutritional agar media and incubated for 5 days at 30 or 37°C, respectively. The *stp2*Δ mutant formed an extended hyphal fringe on nitrogen starvation medium (SLAD) **(A)** or mannitol-containing Spider agar **(B)**. In contrast, hyphae formation was reduced on RPMI medium **(C)**.

Hyphae formation in planktonic liquid cultures and on solid agar surfaces can differ in their manifestation, since they rely on different genetic programs (Azadmanesh et al., 2017). Therefore, a range of solid media and conditions were tested for hyphae formation and radial filamentation, regardless of inner colony wrinkling, was scored (Supplementary Figure S3). The nitrogen-limiting SLAD agar promotes filamentation in *C. albicans* in line with the starvation response. Stp2-defective strain growth on SLAD agar formed longer filaments under all temperatures and pH conditions tested, with a more pronounced effect at pH 4.2 (Figure 5A). This phenotype was exclusively observed on SLAD agar, but not in liquid conditions (Supplementary Figure S4). Incubation on Spider agar induced colony wrinkling in all strains tested. However, while radial filaments were barely seen in the wild type strain, the *STP2*-defective mutant formed a prominent hyphal fringe at the edge of the colony. Interestingly, this effect was rather mild at 37°C, but striking at an incubation temperature of 30°C (Figure 5B). The incubation in liquid Spider medium for 90 min at 37°C revealed more robust filamentation for the wild type compared to the *stp2*Δ mutant (Supplementary Figure S4), pointing toward differential responses to liquid and solid conditions. In contrast, filamentation on solid RPMI medium was reduced in the *stp2*Δ deletion strain regardless of the incubation temperature (Figure 5C). Growth on another typical mammalian cell culture medium, medium M199, brought out a similar phenotype (Supplementary Figure S3). Therefore, Stp2 controls filamentation in a nutrient-dependent manner.

### *Stp2*Δ Mutant Forms Thinner Biofilms Under Flow Conditions

**FIGURE 6:**
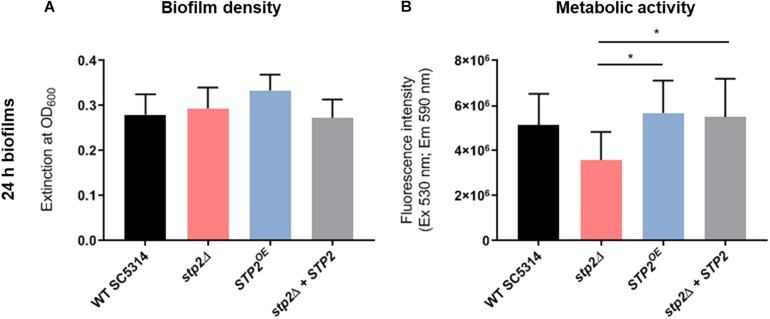
Characterization the role of Stp2 in cell density and respiration of mature *C. albicans* biofilms. *C. albicans* biofilms were grown for 24 h in RPMI on polystyrene plates, then analyzed for cell density by measuring the optical density **(A)** and for respiration of metabolically active cells using resazurin reduction assay **(B)**. *stp2*Δ biofilms were significantly less metabolically active compared to the *STP2* overexpression and gene revertant strains (One-way ANOVA, *p* = 0.0188, *n* = 3).

Maturation from attached monolayers of germ tubes to a dense, matrix-embedded population of cells is the third step of the biofilm development. Thus, we evaluated the effect of Stp2 on biofilm maturation. After the initial adherence phase, the medium was renewed and mature biofilms were allowed to form at 37°C with gentle shaking. Calculation of biofilm formation after 24 h of growth revealed no significant differences for all strains tested, showing that although the *stp2*Δ cells had defects in the early stages of biofilm formation, they were able to reach the density of the Stp2-containing biofilm formers (Figure 6A).

**FIGURE 7:**
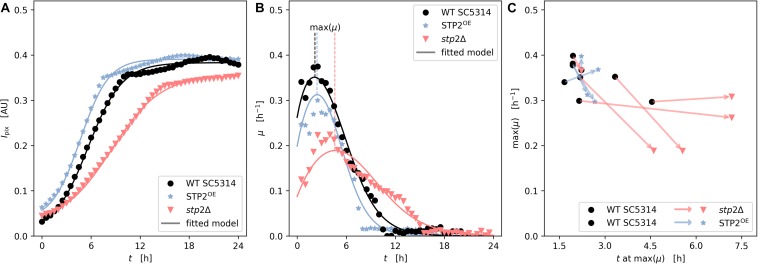
Stp2 plays a crucial role during biofilm formation under flow conditions. Yeast cells were inoculated into a channel system of microfluidic plates and adherence took place for 90 min in RPMI medium without any medium flow. Non-adherent cells were rinsed by a short, but impulsive flush with medium and biofilms were allowed to form under constant slow medium flow. Biofilm architecture and biomass accumulation was monitored microscopically every 20 min over 24 h. **(A)** Time series of measured and modeled cell density (expressed as mean pixel intensity) showing growth over time. **(B)** Time series of measured and modeled specific growth rate representing the relative growth velocity. **(C)** Scatter subplot of height and time of maximal observed growth rate from independent replicates (*n* = 5 for *stp2*Δ vs wild type and *n* = 4 for *STP2*^OE^ vs wild type). The arrows link the maximal growth of *STP2* deletion and overexpression strains (arrow head) to their respective wild types (arrow tail) during one technical approach.

Flow conditions promote *C. albicans* biofilm formation due to the increased availability of nutrients and imposed shear stress (Mukherjee et al., 2009; Uppuluri et al., 2009). Therefore, we applied a microfluidic technology to grow biofilms under flow conditions. After cells were allowed to adhere to the fluidic chamber under static conditions for 90 min the non-adherent cells were rinsed. At this stage the morphology and cell density the *stp2*Δ mutant strain appeared comparable to those obtained from the static cultures, since fewer cells attached to the surface, and those that remained showed a reduced germ tube length (Supplementary Material M1). Next, shear flow of RPMI medium was applied at 0.2 dyne/cm^2^. Biofilm formation was recorded by imaging of the microfluidic chambers every 20 min for 24 h (Supplementary Material M1). The progress of biofilm formation was monitored using an automated computational approach, which analyzed the gray-scale values of the microscopic images (Weise et al., 2020). Based on this, the specific rates of biofilm development were calculated. Interestingly, biofilm maturation differed from static experiments in that the strain lacking *STP2* had an overall slower growth rate, resulting in lower maximal biomass (Figures 7A,B). Moreover, the growth and plateau phase were delayed, and never reached the wild type biofilm confluency as notable differences in biofilm density were detected (Figure 7A). However, hyphae appeared indistinguishable between the two strains at later stages of biofilm development. Biofilm formation by the *STP2* overexpression strain was similar to the wild type under static growth (Figure 6A), and slightly increased under shear flow conditions and (Figure 7). Although some variance within the technical replicates was observed (Figure 7C), the comparative analyses of related measurements revealed a consistent pattern of retarded growth rates for the *stp2*Δ mutant (Figure 7C). The applied analysis not only provided high temporal and spatial resolution of the growing flow-induced *C. albicans* biofilms, but also allowed for the calculation of maximal growth rates and investigation of experimental reproducibility.

### Mature *STP2*-Defective Biofilms Differ in Metabolic Activity and Longevity

**FIGURE 8:**
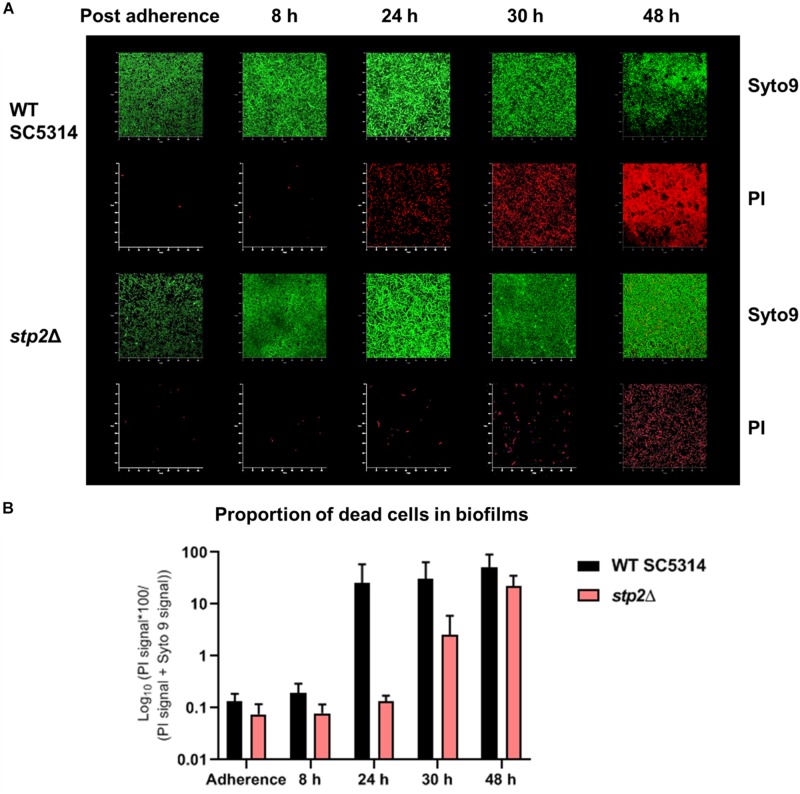
Loss of *STP2* enhances longevity of *C. albicans* biofilms. Biofilms of the *stp2*Δ mutant and wild type were grown on polymer imaging dishes in RPMI medium for up to 48 h. To evaluate cell viability, live-dead staining (Syto9 and PI staining) was applied to single dishes at different time points. Partial cell death of the wild type occurred already after 24 h, whereas cells of the *stp2*Δ mutant biofilm started to die with a large temporal delay at 48 h. **(A)** Exemplary render images of *C. albicans* wild type SC5314 and *stp2*Δ biofilms in time series with live-dead staining in split Syto9 and PI channels. **(B)** The ratio of the red PI signal over the entire cell population (PI + Syto9) was calculated over the z-stack and the median of biological triplicates with SD was plotted in percentage on a log_10_ scale. (*n* = 3).

Although the *STP2* deficient strain was able to reach the density of wild type mature biofilm under static conditions, its metabolic activity was markedly reduced after 24 h of growth (Figure 6B). This phenotype was fully restored in the revertant and overexpression strains. Differences in the total metabolic activity could result from variations in total biomass, energy flux, or the number of live cells. Thus, the viability of cells in wild type and *stp2*Δ biofilms was investigated. At early time points (8 h), nearly all adherent germ tubes or young biofilm cells of the wild type and *stp2*Δ mutant were alive (Figure 8). After biofilm maturation at 24 and 30 h, the wild type biofilms had a large proportion (26 and 31%, respectively) of dead cells (Figure 8). In stark contrast, *stp2*Δ biofilms had enhanced longevity since only a limited number of dead cells were detectable at the same time point. After 2 days the *stp2*Δ biofilms contained about 22% of dead cells, whereas about the half of all wild type biofilm cells were PI-positive. This indicates that absence of the *STP2* results in a notable delay in death of cells within a biofilm. Taken together, although *stp2*Δ mutant formed comparable to the wild type strain static 24 h biofilms, they contained more live cells but with lower metabolic activity.

### Deletion of *STP2* Increases Cellular Sensitivity to the TOR1 Inhibitor Rapamycin

**FIGURE 9:**
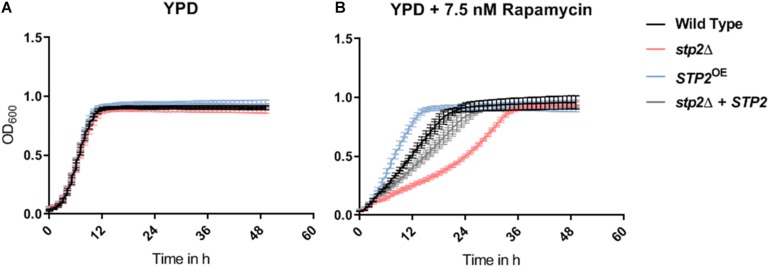
STP2-defective mutants are unrestricted in growth but are suppressed by an antagonist of the TOR pathway. All strains (Wild type SC5314, *stp2*Δ, *STP2*^OE^ and *stp2*Δ + *STP2*) were set to a defined OD_600 nm_ of 0.01 in 200 μl pure and rapamycin-supplemented YPD **(A, B)** media. Incubation was carried out at 30°C and increase of cell density was monitored every 30 min over 2 days (*n* = 3). Although no Stp2-specific effects occurred in standard YPD, the addition of rapamycin to the medium decreased the growth rate of the *STP2* deletion mutant, while increased the growth for the overexpression strain as compared to wild type and revertant strains.

The TOR pathway, active under high levels of intracellular carbon and nitrogen sources, can impact filamentation and adhesion in *C. albicans* (Bastidas et al., 2009). In *S. cerevisiae* TOR controls components of the SPS pathway among others (Shin et al., 2009). Therefore, we investigated whether similar link exists in *C. albicans*. As expected, addition of 7.5 nM rapamycin resulted in a flattened slope of the growth curve in the wild type SC5314 compared to growth in pure YPD medium (Figures 9A,B). The *stp2*Δ mutant exhibited an increased sensitivity to rapamycin during the first 36 h of growth, but eventually reached the cell density of the control strains at the stationary phase. An overexpression of *STP2* increased the growth rate during the early time points, indicating an increased rapamycin resistance. Growth of *stp2*Δ and *STP2*^OE^ strains in pure YPD medium was comparable to the wild type and the complemented strains. Therefore, there was a notable *STP2* gene dosage effect in sensitivity to rapamycin.

## Discussion

The *C. albicans* three component sensor SPS (Ssy1-Ptr3-Ssy5) coordinates the proteolytic cleavage and activation of the transcription factors Stp1 and Stp2 in response to increased levels of exogenous oligopeptides and amino acids, respectively (Martínez and Ljungdahl, 2005; Ljungdahl, 2009; Tumusiime et al., 2011). The proper uptake of amino acids, in particular, is critical for fungal pathogenicity, since the SPS pathway is essential for damage and escape from the macrophages, and for full virulence (Vylkova and Lorenz, 2014; Miramón and Lorenz, 2016; Silao et al., 2019). Sensing and metabolism of certain amino acids, such as arginine, proline and methionine, trigger morphological transition from yeast to hyphae, a well-defined virulence factor. Biofilms represent a microenvironment where morphogenesis and nutrient adaptation are tightly connected. In this study, we investigated how Stp2 drives different stages of biofilm growth. We developed novel approaches for quantitative image analysis and used them to obtain precise measurements of hyphae initiation, biofilm growth rates, and density. Our results show that at the early stage of biofilm formation the *stp2*Δ mutant had impaired adherence to abiotic surfaces and delayed germination, but ultimately formed mature biofilms with density and morphology comparable to control strains. However, *STP2*-deficient biofilms had significantly lower metabolic activity, leading to increased biofilm longevity and sustainability. Taken together, this study demonstrates that *C. albicans* Stp2-coordinated processes are critical for biofilm formation and longevity.

Adherence to surfaces is the initial step of biofilm formation. In *C. albicans* this is mediated by GPI-modified cell wall proteins named adhesins. The induced expression of hyphae-associated adhesins, such as Hwp1 and Als3, during the yeast-to-hyphae switch promotes adhesion to epithelial and endothelia host cells and mature biofilm structure on silicone surfaces (Zhao et al., 2004, 2006; Wächtler et al., 2011). In this study, Stp2 regulated the adherence to abiotic surfaces, as fewer *stp2*Δ mutant cells attached to the bottom of the polystyrene well or the microfluidic channels. Stp2-dependent expression of *HWP1* and *ALS3* was also notable in planktonic cultures, which strengthens the link between amino acids metabolism and adherence. In this regard, a screening of transcription factor library for adherence in YPD under flow condition revealed a number of adhesion-defective mutants that are regulators of amino acid and general nitrogen metabolism (Finkel et al., 2012). These include controllers of branched-chain or sulfur-containing amino acid biosynthesis genes (Leu3, Met4), utilization of ornithine (Arg81), and putative arginine biosynthesis (Dal81) (Finkel et al., 2012), highlighting the importance of amino acid metabolism for adhesion.

Aside from the observed reduction in the number of adherent cells, we noted a marked decrease in germ tube formation in the *stp2*Δ mutant. The recognition and uptake of certain extracellular amino acids induces morphological changes (Lee et al., 1975; Dabrowa et al., 1976; Kraidlova et al., 2011). For instance, Silao et al. (2019) recently discovered that metabolism of proline results in Ras1/cAMP/PKA-mediated hyphae formation. Defects in filamentation were also described for mutants in key components of the SPS pathway, which senses increase in the extracellular level of some amino acids, but not proline. The Ssy1 sensor is important for filamentation on solid Lee’s plates or serum-containing medium (Brega et al., 2004), while Stp2 is required for yeast-to-hyphae switch on ornithine agar (Silao et al., 2019). A reduced ability of the Stp2-deficient strain to initiate hyphal morphogenesis was also observed here - in the context of biofilms, in liquid RPMI, and on solid media. Interestingly, the filamentation defect in liquid medium corresponded to the degree of Stp2 processing – *stp2*Δ fails to filament in amino-acid rich medium (Vylkova and Lorenz, 2014), a strong Stp2-stimulating condition (Miramón and Lorenz, 2016), whereas partial processing in RPMI only caused a germination delay. We hypothesize that the observed lag in hyphal growth in the latter condition is achieved by activation of alternative pathways that react to changes in the intracellular nutritional state, and that such compensatory mechanisms fail in cells with gross growth defects (such as *stp2*Δ cells in CAA). In *C. albicans* the former effects might be driven by the Gcn4-controlled general amino acid control (GAAC) or the TOR pathways, which are linked to the SPS pathway and to each other by mutual control hubs (Garbe and Vylkova, 2019). Indeed, we show that loss of Stp2 results in enhanced expression of *GCN4*, which is regulated mostly at the transcriptional level in *C. albicans* (Tournu et al., 2005), and an increased sensitivity to the Tor1-inhibitor rapamycin, supporting the idea of compensatory nutrient responses by these pathways. A correlation between the Stp1/2 transcription factors and TOR signaling was already described in *S. cerevisiae*, where *stp1*Δ cells were hypersensitive to rapamycin and Stp1 protein degradation was controlled by ScTORC1 (Shin et al., 2009). Since the components of the TOR-complex differ fundamentally between *S. cerevisiae* and *C. albicans* (Tatebe and Shiozaki, 2017), the connections between TOR and members of the SPS pathway could have been rewired.

Several studies revealed a transcriptional upregulation of amino acid uptake and biosynthesis genes in mature biofilms. The basic amino acid permease gene *CAN2* is one of the common core target genes of the master biofilm regulators, and shared a transcriptional response with the hyphae-associated core target genes *ALS1*, *HYR1*, and *HWP1* (Nobile et al., 2012). Rajendran and colleagues compared high and low *C. albicans* biofilm forming isolates in the context of amino acid biosynthesis, and showed that the aspartate aminotransferase Aat1 is a key factor for biofilm heterogeneity (Rajendran et al., 2016). Further, the transcription factor of sulfur amino acid biosynthesis Met4 is required for adherence (Finkel et al., 2012), and its target genes for methionine biosynthesis strongly upregulated in mature biofilms (García-Sánchez et al., 2004). The role of amino acid biosynthesis was further shown by metabolomic analysis, where amino acids were intracellularly enriched during the whole process of *C. albicans* biofilm formation (Zhu et al., 2013). A high number of amino acid biosynthesis genes are regulated by Gcn4, and this transcription factor is required for biofilm formation (García-Sánchez et al., 2004). Interestingly, *gcn4*Δ mutant biofilms have the same proportion of hyphae compared to the wild type (García-Sánchez et al., 2004), in line with our observations of *stp2*Δ mature biofilms. Based on these reports and data presented here, we propose that Stp2-mediated amino acid sensing and uptake is particularly important during the early phase of biofilm formation, whereas the GAAC activation in the maturing biofilm leads to increased amino acid biosynthesis. Consistent with this, constant supply of fresh nutrients under flow, a condition that presumably does not require induction of amino acid biosynthesis, leads to prolonged filamentation defect in the *stp2*Δ mutant. A main question that remains to be answered is why *C. albicans* biofilm growth requires such tight control of amino acid homeostasis.

Many longevity pathways are highly conserved in eukaryotes, including mitochondrial function, TOR and PKA signaling, and metabolic pathways such as NAD^+^ biosynthesis (Lin and Austriaco, 2014). Although the exact mechanisms for how these factors coordinate longevity are yet to be discovered, it is now well established that nutritional restriction enhances lifespan in many eukaryotes. In *S. cerevisiae*, for example, the process is mediated through the Tor1-Sch9 and Ras2-cAMP-PKA pathways (Longo, 2003; Deprez et al., 2018), whereas in *C. albicans* low glucose levels were able to extend the lifespan of *goa1*Δ mutant, which has impaired mitochondrial function (Chen et al., 2012). Cells lacking *STP2* formed mature biofilms with reduced metabolic activity compared to the control strains. Thus, failure to take up amino acids likely leads to intracellular nutrient deficiency, which could result in lowered nutrient consumption rates. In this study, we observed a plethora of dead wild type cells after 24 h of biofilm formation, whereas the cell death was notable first in 30 h *stp2*Δ biofilms, indicating increased survival rate. Although calorie reduction is often investigated in form of glucose depletion, a role of amino acid limitation was demonstrated in *S. cerevisiae*: high Gcn4 activation was sufficient to repress protein synthesis, which further increased yeast lifespan and longevity (Steffen et al., 2008). Rapamycin enhanced the Gcn4-associated lifespan extension and intracellular proline accumulation extended the replicative lifespan, suggesting that cellular amino acid homeostasis is critical for yeast aging (Mittal et al., 2017; Mukai et al., 2019). Indeed, failure to properly sense and uptake extracellular amino acids, such as in the *S. cerevisiae* SPS signaling cascade deletion mutants, resulted in enhanced longevity and replicative lifespan under both rich and calorie restricted growth conditions (Tsang et al., 2015), a phenotype that is similar to the longevity effect of *stp2*Δ biofilms described here. Further studies are required to characterize the regulators of lifespan in *C. albicans* and the contribution of this process to adaption and survival in different body niches and fungal pathogenicity.

Overall, our data describe a novel role for *C. albicans* transcription factor Stp2 in adherence, germ tube initiation, and biofilm sustainability, showing that the SPS-mediated responses to extracellular amino acids are critical for biofilm development. In this setting, amino acids not only represent an important alternative nutrient source, but also coordinate hyphal growth, fitness, and biofilm lifespan. The connection between Stp2-regulated processes to pathways that control intracellular nutritional homeostasis points toward a larger regulatory network that remains to be investigated further.

## Materials and Methods

### Strains and Culture Conditions

*C. albicans* strains were routinely passaged on YPD agar (2% peptone, 1% yeast extract, 2% glucose, 1.5% agar) at 30°C and stored as frozen stocks in YPD medium using Roti^®^-Store yeast cryovials (Carl Roth GmbH + Co. KG). RPMI with stable glutamine (Merck–Biochrom) was used as induction media for *in vitro* biofilm and planktonic cultures assays. Liquid CAA medium (0.17% YNB, 0.5% ammonium sulfate and 1% casamino acids; pH 4.5) was used to trigger Stp2 activation, while SD medium (0.17% YNB, 0.5% ammonium sulfate and 2% glucose; pH 4.5) was used as synthetic minimal medium that does not stimulate the SPS-pathway. *C. albicans* strains used in this work are listed in Table 1.

**TABLE 1:** *C. albicans* strains in this study.

**Name**	**Genotype**	**References**
Wild type SC5314	wild type	Fonzi and Irwin, 1993
*stp2*Δ	*stp2Δ::FRT/stp2Δ::FRT/stp2Δ::FRT*	Vylkova and Lorenz, 2014
*stp2*Δ+*STP2*	*stp2Δ::FRT/stp2Δ::FRT/stp2Δ::FRT-STP2*	Vylkova and Lorenz, 2014
*STP2*^OE^	*ADH1/adh1::STP2-SAT1*	this study
*STP2-TAP*	*stp2Δ::FRT/stp2Δ::FRT/stp2Δ::FRT RPS1/rps1::TDH3p-STP2-TAP-SAT1*	this study

### Growth Assays

General proliferation was evaluated via growth curve assays. Strains were pre-cultured overnight in YPD at 30°C and diluted to OD_600 nm_ of 0.01 in different growth media (YPD, YPD with 7.5 nm rapamycin, RPMI, RPMI with 0.5% ammonium sulfate). Cultures were incubated at 30°C in a Magellan TECAN plate reader and OD_600 nm_ determined prior 30 s shaking every 30 min over 48 h. Changes in cell density (OD_600 nm_) were plotted in biological triplicates against the incubation time.

### Strain Construction

#### Plasmid Construction

For the ectopic overexpression of *STP2*, the previously described pADH1-GFP (Hünniger et al., 2014) was used. *STP2* was amplified with the primer pair 5’STP2-XhoI and 3’STP2-EcoRV and cloned via *Xho*I/*EcoR*V into the plasmid, thereby replacing *GFP*. The created plasmid was named pSTP2OE_w/o_TAG.

Tagging of *STP2* was done using the Gateway^TM^ cloning approach (Legrand et al., 2018). The *STP2* gene was amplified from genomic DNA with the primer pair STP2_Gateway_fwd and STP2_Gateway_rev and subsequently cloned into pDONR221^TM^ (Thermo Fischer) via BP Clonase^TM^ reaction. The resulting *STP2* donor vector was added to a LR Clonase^TM^ reaction with the expression vector pDEST2303 (Legrand et al., 2018) to generate an expression plasmid carrying *STP2* with a C-terminal TAP-tag under control of the *TDH3* promoter and a *SAT1* selection marker. The created plasmid was named pDEST2303_(STP2).

#### *C. albicans* Transformation

For the integration of *STP2* in the Ca*ADH1* locus, a transformation cassette was created via *Nar*I/*Sac*I-restriction of pSTP2OE_w/o_TAG, while pDEST2303_ (STP2) was digested with *Stu*I for integration in the Ca*RPS1* locus. The resulting transformation cassettes were purified via gel extraction (QIAquick Gel Extraction Kit, Qiagen) and used for *C. albicans* transformation using the previously described lithium-acetate-method (Walther and Wendland, 2003). After the heat shock, the cells were incubated for 4 h in YPD at 37°C prior to plating on YPD agar plates containing 200 μg/ml nourseothricin (Werner BioAgents). Transformants were validated via colony PCR. The validation primers are listed in Supplementary Table S1.

### Morphological and Biofilm Tests

#### Germ Tube Assay

For hyphae induction in liquid medium, *Candida* strains were pre-cultured in liquid YPD overnight (30°C, 180 rpm), washed with PBS, and stained for 30 min with 1% rhodamine and 0.05% Tween 80 in the dark. The cells were washed twice with PBS and a final cell density of OD_600 nm_ of 0.4 (8 × 10^6^ cells) was transferred into 500 μl RPMI induction medium for 90 min cultivation at 37°C. After germ tube induction, the cells were counterstained with 2% calcofluor white for 5 min and the morphology of the washed samples was analyzed microscopically (Axiovert, Carl Zeiss, AG). The experiment was performed in biological quadruplicates.

#### Filamentation Assay

*Candida* cells from YPD overnight cultures were washed and 5 μl cell suspensions (OD_600 nm_ of 0.5) were spotted on the following agar plates (2% Kobe I agar, Carl Roth GmbH + Co., KG): YPD; Spider (1% nutrient broth, 1% mannitol, 0.2% K_2_HPO_4_); SLAD (synthetic low ammonium dextrose: 0.17% YNB w/o amino acids and w/o ammonium sulfate, 2% glucose, pH 4.2 and pH 7); M199 (without phenol red, pH 4 and pH 7, from Sigma-Aldrich Chemie GmbH); YNB + 2% Glucose; YNB + 2% Glucose + 10% fetal bovine serum (ZellBio GmbH); YNB + 2% N-acetylglucosamine (Sigma-Aldrich Chemie GmbH) or RPMI (Merck–Biochrom, liquid medium supplemented with water agar). Colony filamentation was investigated after incubation at 30°C or at 37°C in either atmospheric or 5% CO_2_-enriched air after 5 days incubation using a binocular (Stemi 305, Carl Zeiss AG). Radial filamentation was scored from 0 (yeast) to 4 (elongated hyphae). Filamentation assays in liquid media were performed in Spider, M199 (pH 4 and pH 7) and SLAD (pH 4.5 and pH 7) media. The composition of the liquid media was equivalent to the solid media, except the agar and the addition of 50 μM ammonium sulfate to the SLAD media. *Candida* cells from YPD overnight cultures were washed, transferred into 500 μl induction media (OD_600_ nm of 0.1) and incubated for 90 min at 37 °C under constant shaking (180 rpm). The morphology was investigated microscopically scored from 0 (yeasts only) to 4 (true hyphae only).

#### Adherence Assay

Assays for *C. albicans* adherence and biofilm formation were adapted from established protocols [summarized in Gulati et al. (2018)]. Briefly, *C. albicans* cells were pre-grown in YPD overnight and PBS washed cells were set to OD_600 nm_ of 0.5 in 1 ml RPMI medium in 24 well plates (Costar^®^, Corning^®^). For microscopic analyses, cells were set to a lower density (OD_600 nm_ of 0.05) and seeded in microscopy-optimized 24 well plates (μ-Plate 24 Well Black, IBIDI). Cells were allowed to adhere for 90 min at 37 °C under constant shaking (100 rpm). Non-adherent cell were washed three times with cold PBS and the density of the remaining cells was measured by extinction at OD_600 nm_. Cell morphology was assessed microscopically.

#### Biofilm Assay

The first steps of biofilm assay are identical to the adherence protocol starting with OD_600 nm_ of 0.5 cultures, with the cell staining steps omitted. Following cell adherence, 1 ml of pre-warmed RPMI medium (or the indicated medium) was added and biofilms were formed for 24 h at 37°C and 100 rpm. Next, supernatants were discarded, and biofilms were washed once with PBS. Biofilms were quantified for cell density (OD_600 nm_) and metabolic activity with the help of resazurin reduction assays. For this, biofilms were resuspended in 500 μl PBS plus 50 μl of resazurin (0.15 mg/mL) and incubated at 37°C for 1hr in the dark. Fluorescent intensity (excitation 530 nm and emission 590 nm) of the reduced product resorufin was measured in a TECAN plate reader.

#### Biofilm Assay Under Flow Condition

Biofilm formation under shear flow conditions was monitored using the Bioflux1000 device (Fluxion Biosciences, Inc.) following established protocols (Gulati et al., 2017). In short, *C. albicans* overnight cultures were washed and set to OD_600 nm_ of 0.5 in pre-warmed RPMI medium. Cells were seeded with 2 dyne/cm^2^ (0.2 Pa) for 2–5 s from the outlet well into the channels of Bioflux1000 48 well plates, which were primed before with warm medium. The cells were allowed to adhere to the channels for 90 min without any flow (0 dyne/cm^2^), followed by removal of non-adherent cells by flowing fresh, pre-warmed RPMI medium with 2 dyne/cm^2^ for 5 s. Shear flow was set to 0.2 dyne/cm^2^ for time series experiments over 24 h biofilm formation and images were captured every 20 min. Two channels were investigated in parallel having a 10 × magnification to allow a direct comparison between a mutant and a reference strain. Image capturing and stacks to movies was performed using the MetaMorph^®^ software (Molecular Devices).

#### Three-Dimensional Analysis of the Biofilm Morphology and Vitality

The general biofilm protocol was adapted for high resolution imaging as follows: cells were seeded with OD_600 nm_ of 0.5 in 2 ml colorless RPMI in imaging dishes (μ-Dish 35 mm, high and uncoated, IBIDI) and adherent cells and maturing biofilms were stained for alive and dead cells using the Filmtracer kit (Filmtracer^TM^ LIVE/DEAD^TM^ Biofilm Viability Kit, Invitrogen^TM^). Syto9 and propidium iodide were applied at a 1:2000 dilution. Z-stack were collected with confocal laser scanning microscopy (CLSM, LSM 780, Carl Zeiss AG) at 1.0 μm intervals and the images were compiled to generate three-dimensional renderings. All confocal parameters were used as standard settings for comparison to the wild type and mutant biofilms produced at different time points. CLSM z-stack processing was performed using the ZEISS ZEN black edition.

### Gene Expression Analyses

*Candida* overnight cultures were set to OD_600 nm_ of 0.3 in 10 ml fresh YPD and were incubated at 37°C, 180 rpm until they reached OD_600 nm_ of 1. Logarithmic cultures were harvested and washed in RPMI prior to the transfer in 50 ml RPMI (OD_600 nm_ of 0.3) and incubation for 60 min (37°C, 180 rpm). Sampling from CAA medium was done similarly except the differences in culture volumes (50 ml YPD medium for logarithmic cultures and 30 ml of a CAA culture). Shock frozen cell pellets were used for RNA isolation by a phenol-chloroform method previously described by Martin et al. (2011). A BioAnalyzer instrument (Agilent) was used to measure RNA quality and RNA concentration was determined via NanoDrop (Thermo Fisher Scientific). Finally, a total amount of 100 ng RNA was used for each qRT-PCR reaction that included SYBRGreen^®^ as fluorescent dye (Brilliant II SYBR Green qPCR Master Mix, Agilent). The experiments were performed in a thermal cycler (Stratagene MxPro-mx3005P, Agilent) and run in biological and technical triplicates. The indicated expression rates are relative to the expression values of the housekeeping gene *ACT1* following the mathematical model from Pfaffl (2001). All primers are listed in Supplementary Table S1.

### Protein Extraction and Western Blot Analysis

To visualize *STP2* processing, *stp2*Δ and *STP2-TAP* were incubated overnight in YPD at 37°C, washed twice with sterile H_2_O, and then set to OD_600 nm_ of 0.3 in 10 ml of SD, CAA or RPMI medium. The samples for RPMI were collected after 90 min, and for SD and CAA after 240 min. The cell pellets were immediately frozen in liquid nitrogen. For extraction of total protein the cell pellets were dissolved in 500 μl of protein extraction buffer (50 mM Tris-HCl, 150 mM NaCl, 0.1% Triton X-100, 1mM DTT, 10% Glycerol) with proteinase inhibitor (Halt Proteinase Inhibitor, 100 μl per 10 ml buffer, Life Technologies) and an equivalent amount of ice-cold glass beads. The cells were then disrupted via vortexing for 10 × 1 min with 1 min recovery breaks on ice in between. Subsequently, the supernatant was transferred into a fresh tube after 10 min centrifugation and the protein concentration measured via BCA assay (Thermo Fischer). A total amount of 7 mg protein was then loaded onto a SDS-gel (Novex^TM^ 10% Tris-Glycine Mini Gels, Thermo Fischer) and run for 1 h with 100 V. The proteins were then transferred onto a PVDF membrane via western blot for 1 h with 100 V, the membrane blocked in 5% milk and cut. Tdh3 was detected as a loading control using the Tdh3-antibody GT239 (also Anti-GAPDH, GeneTex) with Donkey Anti-Mouse DyLight549 (Jackson Immuno Research) as the secondary antibody. *STP2-TAP* was detected with the *TAP* tag polyclonal Antibody CAB1001 (Invitrogen) and Goat Anti-Rabbit HRP (Invitrogen) as the secondary antibody.

### Automated Image Analyses

#### Automated Determination of Hyphal Length in RPMI Medium

Multichannel z-stacks of differentially stained *Candida* cells were first pre-processed with the software Fiji v1.51n (Schindelin et al., 2012). For both channels, stained either with rhodamine (cell bodies) or calcofluor white (cell bodies and hyphae), edge detection was applied and the variance of pixel intensity values of each slice in the z-stack was measured to find the slice with best focus, which is expected to have the highest variance. The rhodamine channel was shifted in x-direction by 1 pixel and in y-direction by two pixels to compensate for an offset between the two channels and resulting black image borders were removed by image cropping. The *Despeckle* function was applied to reduce noise and background subtraction with a rolling ball radius of 250 pixels was performed to homogenize illumination. The rhodamine channel was then exported for segmentation of yeast cell bodies.

Yeast cell detection and splitting of cell clumps was implemented in Python v3.7 using the computer vision library OpenCV (Bradski and Kaehler, 2000). Pixel values of pre-processed rhodamine images were first scaled to a range from 0 to 255. Adaptive thresholding with a Gaussian window and block size of 21 pixels was performed to binarize images. Foreground objects with an area less than 30 pixels were removed to omit small objects that are not expected to be yeast cells. Cell regions missed by adaptive thresholding were filled by applying a global threshold set at 1.3 times the threshold value obtained by Otsu’s method and adding the result to the previous segmentation result. Objects with solidity less than 0.6 were removed and holes of remaining objects were filled. Hyphal structures were then removed by morphological opening with a circle of 7 pixels diameter. Splitting of cell clumps was performed as described by Brandes et al. (2017) with parameters set to *n* = 5, *m* = 3, *min*_dst_ = 3, *min*_area_ = 60, *l*_min_ = *n*, *w*_min_ = *m*, *l*_max_ = 5*n*, *w*_max_ = 5*n*, and *shift* = 3. In brief, contours of foreground objects containing at least *min*_area_ pixels were scanned in intervals of *shift* pixels for concavity points using chords of length *n*. Only concavities with a depth of at least *min*_dst_ pixels were taken into account. A dynamic window with minimum length *l*_min_ and width *w*_min_ and maximum length *l*_max_ and width *w*_max_ was used to find split lines between identified concavity points. Resulting split lines were then refined in a post-processing step and small objects with an area less than 5 pixels, created during cell clump splitting, as well as objects larger than 2000 pixels were removed. Additionally, only objects with a mean intensity value of at least 20 were kept to remove remaining artifacts with intensities lower than yeast cells. The final binary objects were used as a mask for hyphal length quantification.

Hyphal lengths were measured in Fiji. First, the calcofluor white channel was smoothed by convolution with the edge-preserving Kuwahara filter with a sampling window width of 3 pixels. A global threshold value was obtained by multiplying the value of Li’s autothreshold with factor 1.5 and used to binarize the image. Small artifacts were removed by applying the *Remove Outliers* function with *radius* = 2 and *threshold* = 50. All remaining foreground objects were detected using the *Analyze Particles* function excluding objects that touch the image border and images with more than 500 objects were skipped to avoid length measurement inaccuracies due to crossing hyphae. For the remaining images, the number of yeast cells present in each foreground object was counted based on the yeast cell mask image and only objects containing one cell were taken into account to avoid crossing hyphae in cell clumps. The mask image was dilated two times and subtracted from the skeletonized objects image to obtain an image containing only hyphae with one pixel thickness. The function *Analyze Skeleton (2D/3D)* was then used to find the largest branch for each skeleton, omitting smaller artifacts that are unwanted concomitants of object skeletonization. All remaining skeletons with a length smaller than 2 μm were removed to exclude remaining artifacts that are probably no hyphae. The average hyphal length and distribution of hyphae per cell were exported for each image stack.

#### Images Analyses to Determine Biofilm Growth Under Flow Condition

Computations regarding image pre-processing, image analysis and modeling were performed using the programming language python Python v3.6.9. Source material provided as AVI files was converted into single TIFF images as well as data frames containing meta data annotations. The individual image contains two growth chambers (wild type and mutant) separated by four edge lines. Images were rotated automatically to vertical alignment in order to carry out an automated chamber detection and analysis. The mean pixel intensity (i.e., gray scale value; reflecting cell density) of the individual chamber was calculated and added into the respective data frame. An ODE model reflecting logistic growth as well as a lag phase was fitted to the individual experiments. Fitting was carried out by minimizing a cost function (unweighted least-squares-based) using the *Nelder-Mead*-algorithm (Nelder and Mead, 1965). Growth rate time series generated from the fitted model were used to compare wild type and mutant regarding the maximum observed growth rates as at their respective time points.

Detailed protocols regarding the above analyses can be found under: (Weise et al., 2020)

#### Quantification of Proportion of Dead Cells of Filmtracer-Stained Biofilms

The original 4D (X,Y,Z, color) confocal images were saved and provided for analysis in the native CZI (Carl Zeiss Image) format. The LIVE/DEAD staining technique provided two color channels for each z-stack: one for red (PI staining of dead cells) and one for green (Syto9 staining of live cells) labeling. The death ratio analysis was carried out on by a custom-written Fiji macro, using ImageJ version 1.52 s (Schindelin et al., 2012; Rueden et al., 2017). The preprocessing and analysis parameters were adjustable via an easy-to-use graphical user interface (GUI), also written in the Fiji macro language. The workflow of the death ratio calculation was as follows:

(i) Each channel was assigned its color (red or green) via the GUI;
(ii) Optionally the top and bottom of the z-stack was trimmed to avoid having very low signal to noise ratio (SNR) images to interfere with the reliability of the results. The number of Z layers to be cut off from the top or the bottom was controlled via the GUI;
(iii) The channels were lightly smoothed by a Gaussian filter, using a 2-pixel wide window;
(iv) Outliers were removed from the smoothed images on a per-channel basis, the size limit was adjusted via the GUI. This step served the purpose of removing salt-and-pepper type noise from the images, to further increase SNR. The size limit was adjustable for each channel independently, but in practice the same values (two pixels) were used in the entire analysis.
(v) The red and green channels were thresholded using the Otsu algorithm (Otsu, 1979) as provided by Fiji.
(vi) The foreground pixels were counted for both the red and green channels and the ratio of red/(red + green) was calculated to characterize the share of dead cells in the entire cell population.

All calculations in steps (i) to (vi) were carried out on a per-layer basis in the z-stacks for both channels. The results of the ratio calculations were saved in text files using the CSV format and provided for further analysis. Images from biological triplicates were analyzed and the median values were used to calculate the ratio of dead cell over a z-stack.

## Data Availability Statement

All datasets generated for this study are included in the article/Supplementary Material.

## Author Contributions

BB, EG, and PB designed and performed the wet lab experiments and analyzed the data. BH, TW, ZC, and SD analyzed the image data and derived the models. BB, BH, TW, and EG designed the figures. MF, DD and SV discussed the results and supervised the project. BB, and SV wrote the manuscript in consultation with BH, EG, PB, TW, SD, DD, and MF.

## Conflict of Interest

TW and DD were employed by BioControl Jena. The remaining authors declare that the research was conducted in the absence of any commercial or financial relationships that could be construed as a potential conflict of interest.
