# Supplementary material for: The Transcription Factor Stp2 Is Important for Candida albicans Biofilm Establishment and Sustainability
Source: Front Microbiol. 2020 Apr 30;11:794. doi: 10.3389/fmicb.2020.00794 (PMC7203782; doi:10.3389/fmicb.2020.00794)
Supplement: Supplementary file 1 [file Presentation_1.pdf]

Supplemental data

Figures

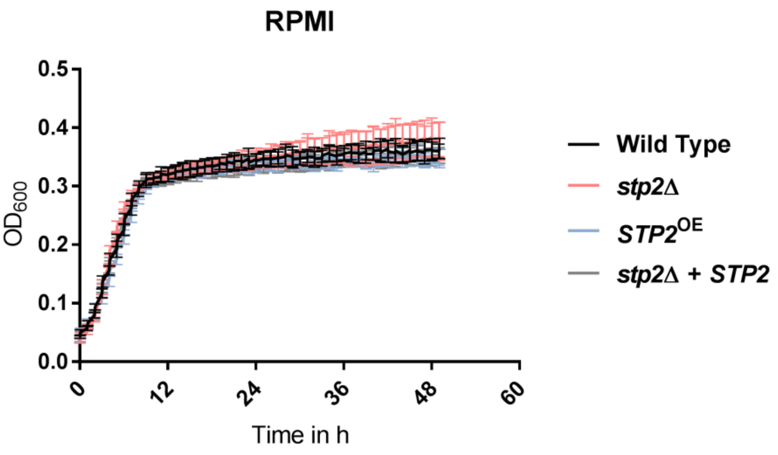

Figure S1 Stp2 does not affect growth in RPMI

All strains (Wild type SC5314, *stp2*Δ, *STP2*^{OE} and *stp2*Δ + *STP2*) were set to a defined $OD_{600\ nm}$ of 0.01 in 200 µl RPMI medium. Incubation was carried out at 30°C in a 96 well plate format and increase of cell density was monitored every 30 min over two days (n = 3). No growth differences were observed.

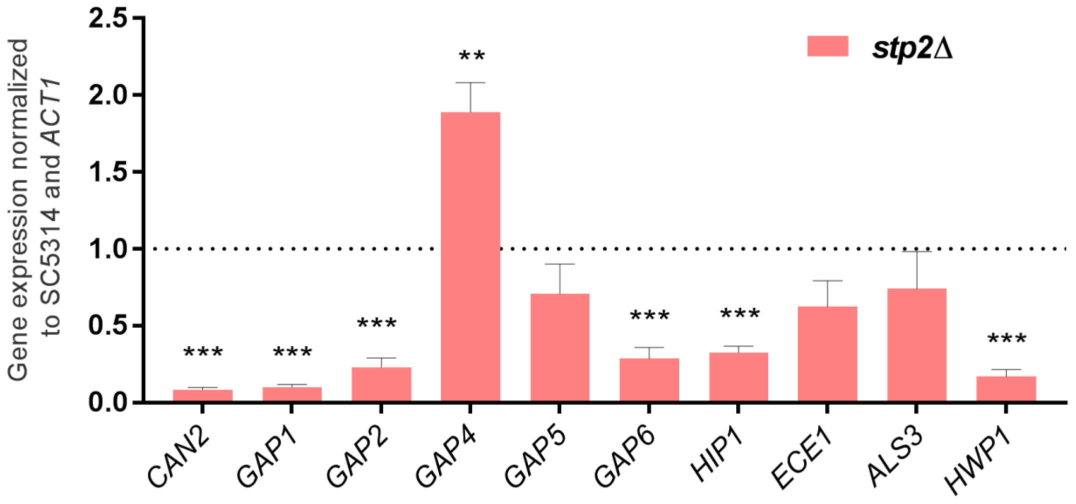

Figure S2 Stp2 activation is required for the expression of hyphae-associated genes

Wild type and the *stp2*Δ mutant were cultured in liquid CAA medium at 37°C for 60 min and the total RNA extracted. Relative expression of amino acid permease and hyphae-associated genes was analyzed using quantitative real time PCR. Depicted bars represent the relative fold expression change normalized to the housekeeping gene *ACT1* and the wild type SC5314 gene expression using the $2^{-\Delta\Delta CT}$ method (Pfaffl, 2001). Shown are results means SD from three biological and three technical replicates. Statistical analyses compared each sample to the SC5314 wild type level (Multiple unpaired t-test, *** $p < 0.001$; ** $p < 0.005$ and * $p < 0.01$; for samples that passed the False Discovery Rate approach by the Benjamini–Hochberg procedure with 1% threshold).

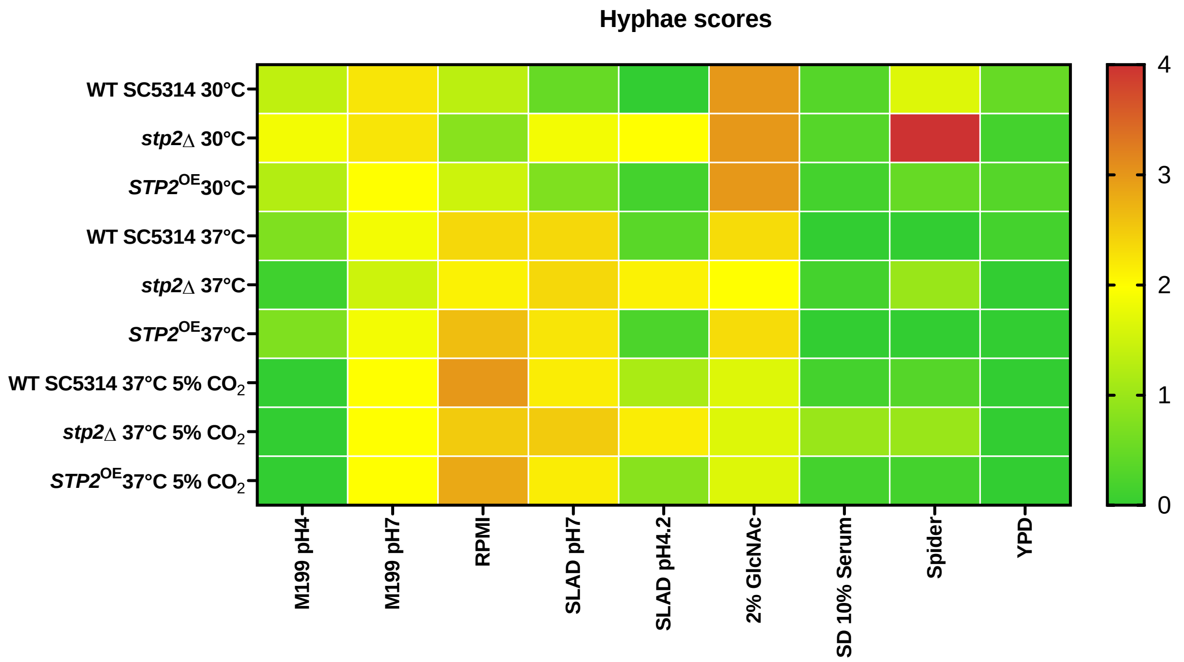

Figure S3 Overview of filamentation ability of *C. albicans* SC5314, *stp2*Δ and *STP2*^{OE} on solid agar

Three *C. albicans* strains were evaluated for filamentation on a number of different nutritional media (see material and methods) after incubation at 30°C, 37°C or 37°C in a 5% CO_2-enriched atmosphere. The ability to form peripheral filaments was scored from 0 = yeast up to 4 = elongated hyphae. The experiment was run in biological triplicates and mean score was plotted as a heat map.

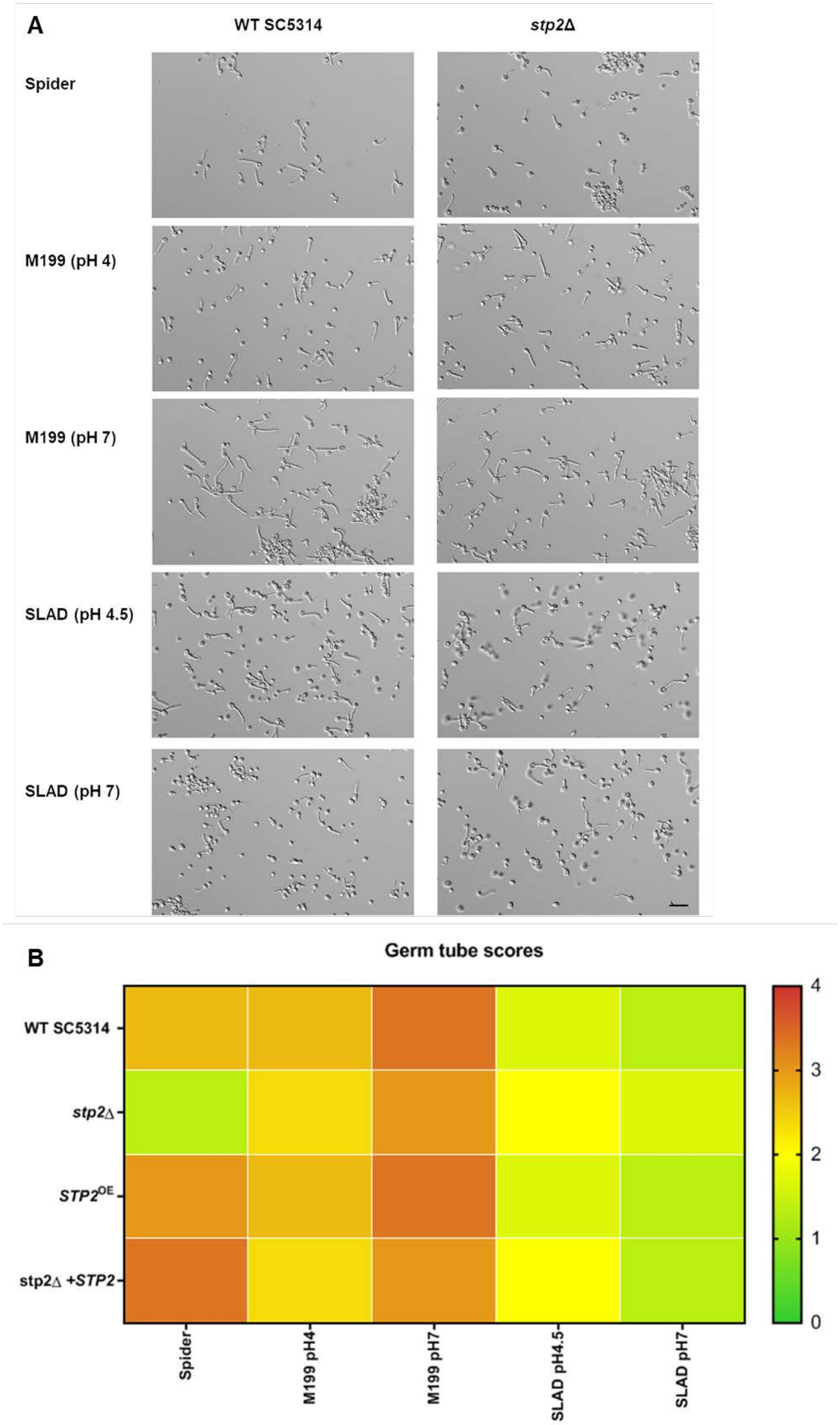

A
WT SC5314
stp2Δ
Spider
M199 (pH 4)
M199 (pH 7)
SLAD (pH 4.5)
SLAD (pH 7)
B
Germ tube scores
WT SC5314
stp2Δ
STP2OE
stp2Δ +STP2
Spider
M199 pH4
M199 pH7
SLAD pH4.5
SLAD pH7
4
3
2
1
0

Figure S4 Overview of filamentation ability of *C. albicans* SC5314, *stp2*Δ, *STP2*^{OE} and *stp2*Δ + *STP2* in liquid media

Filamentation of *C. albicans* SC5314, *stp2*Δ, *STP2*^{OE} and *stp2*Δ + *STP2* strains was evaluated in liquid media (Spider, M199 pH 4 and pH 7 and SLAD pH 4.5 and pH 7) for 90°C incubation at 37°C. A) Exemplary snapshots of the wild type and the *stp2*Δ mutant after germ tube induction of 90 min in the indicated media (Scale bar: 20 µm). B) The ability to form true hyphae was scored from 0 = yeast cells only up to 4 = true hyphae only. The experiment was run in biological triplicates and mean score was plotted as a heat map.

Movie S1 Growth of *C. albicans* in a flow chamber

The Bioflux 1000 device was used to monitor the development of *C. albicans* biofilms under constant shear flow. Yeast cells were diluted in RPMI medium and seeded into the channel system of a Bioflux 48 well plate. The attachment phase was performed for 90 min at 37°C without shear flow and non-adherent cells were washed away by a medium pulse. The growth and morphogenesis were followed microscopically over 24 h and time-serial images were stacked. The *stp2*Δ mutant was less adherent compared to the wild type, because fewer cells remained attached to the channel after the removal of non-adherent cells. Although both strains started to filament rapidly with a strong hyphae cross-linkage, the mutant strain was impaired in biofilm formation and never reached the biomass of the wild type.

Table S1 Primers used in this study

Restriction sites for cloning approaches were indicated in lowercase.

Primer name	Application	Sequence
STP2-R1	qrtPCR	ATT GCC ACT CCA GCA TCG A
STP2-R2	qrtPCR	AACATCAAGACAATATTCATCGTC
ACT1-R1	qrtPCR	TCAGACCAGCTGATTTAGGTTTG
ACT1-R2	qrtPCR	GTGAACAATGGATGGACCAG
ECE1-R1	qrtPCR	ATCGAAAATGCCAAGAGAG
ECE1-R2	qrtPCR	AGCATTTTCAATACCGACAG
HWP1-R1	qrtPCR	ATCAGCTCCTGCCACTGAAC
HWP1-R2	qrtPCR	TGAGTGGAACTGATTCTAATGTAGTTG
GCN4_R1	qrtPCR	AAAGTTGATCATTTGGGTTG
GCN4_R2	qrtPCR	ATTCTTTCCATTTTACGAGC
R1-BCR1	qrtPCR	ACTTTACCCCCAGTATCAAGCA
R2-BCR1	qrtPCR	ATCCAGTTTATTCACTACAACCATAG
R1-CaALS3	qrtPCR	ATGGTCCTTATGAATCACCATCTA
R2-CaALS3	qrtPCR	TAGCAGTTGTAGTTGTAGATGGAG
R1 CAN2_2	qrtPCR	TTGTTCACTGCGGCATTTGG
R2 CAN2_2	qrtPCR	TCCCCAAGCGATCAATCCAG
R1 GAP1	qrtPCR	TGCCTTAATTGCTGCTGATA

R2 GAP1	qrtPCR	AAATATGATCCCCAAACCCC
HIP1-R1_2	qrtPCR	ATGTCACGTCCGATTCAGG
HIP1-R2_2	qrtPCR	GATTAAAATGACAACTGCTCCCAA
5'STP2-XhoI	cloning	TCAGctcgagATGTCAGTAGCAATAACATCCAATA
3'STP2-EcoRV	cloning	AGCTgatatcTCAGAAATACATTGATGGTTGTTGT
STP2-veri_rev	colony-PCR	ACGAACTCTAAACTCGGCATC
G1-ADH1	colony-PCR	TATTCCGGAAGCTGGTAGCG
G2-ADH1	colony-PCR	CCTAGTTGCCCTCCTTATGA
I1-SAT1	colony-PCR	CGGTGATCCCTGAGCAGGTGGCG
CaACT1term veri rev	colony-PCR	GAATACAAAACCAGATTTCCAGATTTCCAG
STP2 veri	colony-PCR	CCATCTTAAACTGCGTCATTTCAATTA
